# Control of ventricular excitability by neurons of the dorsal motor nucleus of the vagus nerve

**DOI:** 10.1016/j.hrthm.2015.06.005

**Published:** 2015-11

**Authors:** Asif Machhada, Richard Ang, Gareth L. Ackland, Natalia Ninkina, Vladimir L. Buchman, Mark F. Lythgoe, Stefan Trapp, Andrew Tinker, Nephtali Marina, Alexander V. Gourine

**Affiliations:** * Centre for Cardiovascular and Metabolic Neuroscience, Department of Neuroscience, Physiology and Pharmacology, University College London, London, United Kingdom; † Centre for Advanced Biomedical Imaging, University College London, London, United Kingdom; ‡ William Harvey Heart Centre, Barts & The London School of Medicine and Dentistry, London, United Kingdom; § Department of Medicine, University College London, London, United Kingdom,; ¶ School of Biosciences, University of Cardiff, Cardiff, United Kingdom

**Keywords:** **7-NI**, 7-Nitroindazole, **AlstR**, allatostatin receptor, **AVNERP**, atrioventricular node effective recovery period, **DVMN**, dorsal vagal motor nucleus, **eGFP**, enhanced green fluorescent protein, **LVV**, lentiviral vector, **NO**, nitric oxide, **nNOS**, neuronal nitric oxide synthase, **QTc**, corrected QT interval, **SNRT**, sinus node recovery time, **vERP**, ventricular effective refractory period, **VT**, ventricular tachycardia, **WT**, wild type, Arrhythmia, Atrioventricular, Brain, Cardiac electrophysiology, Nitric oxide, parasympathetic, Parkinson’s disease, Vagus nerve, Ventricle

## Abstract

**Background:**

The central nervous origins of functional parasympathetic innervation of cardiac ventricles remain controversial.

**Objective:**

This study aimed to identify a population of vagal preganglionic neurons that contribute to the control of ventricular excitability. An animal model of synuclein pathology relevant to Parkinson’s disease was used to determine whether age-related loss of the activity of the identified group of neurons is associated with changes in ventricular electrophysiology.

**Methods:**

In vivo cardiac electrophysiology was performed in anesthetized rats in conditions of selective inhibition of the dorsal vagal motor nucleus (DVMN) neurons by pharmacogenetic approach and in mice with global genetic deletion of all family members of the synuclein protein.

**Results:**

In rats anesthetized with urethane (in conditions of systemic beta-adrenoceptor blockade), muscarinic and neuronal nitric oxide synthase blockade confirmed the existence of a tonic parasympathetic control of cardiac excitability mediated by the actions of acetylcholine and nitric oxide. Acute DVMN silencing led to shortening of the ventricular effective refractory period (vERP), a lowering of the threshold for triggered ventricular tachycardia, and prolongation of the corrected QT (QTc) interval. Lower resting activity of the DVMN neurons in aging synuclein-deficient mice was found to be associated with vERP shortening and QTc interval prolongation.

**Conclusion:**

Activity of the DVMN vagal preganglionic neurons is responsible for tonic parasympathetic control of ventricular excitability, likely to be mediated by nitric oxide. These findings provide the first insight into the central nervous substrate that underlies functional parasympathetic innervation of the ventricles and highlight its vulnerability in neurodegenerative diseases.

## Introduction

Sudden cardiac death is devastating for the family affected and represents a significant public health burden.^1^ Sudden circulatory collapse is often attributable to malignant arrhythmias, such as ventricular tachycardia (VT). Predictors of sudden cardiac death include conventional coronary risk factors and conditions such as congestive heart failure, as well as markers of parasympathetic (vagal) dysfunction that include reduced heart rate variability and reduced baroreflex sensitivity.^2^, ^3^ It is not surprising that vagus nerve stimulation is being explored as a therapeutic option in heart failure, and one component of its potential benefit may be an antiarrhythmic effect.^4^, ^5^

Experimental studies in animal models have demonstrated profound antiarrhythmic effects of vagus nerve stimulation.^6^, ^7^, ^8^ It effectively reduces the restitution slope, prevents alternans, and increases the ventricular effective refractory period (vERP).^6^ Vagus nerve stimulation also decreases the incidence of ventricular arrhythmia associated with heightened sympathetic activity during myocardial infarction.^8^ There is clinical evidence that vagal tone suppresses an accelerated ventricular rhythm,^9^ and experimental data show that the vagal influence on ventricular refractoriness and arrhythmia threshold is tonic and could be abolished by bilateral vagotomy or systemic muscarinic receptor blockade.^10^ Studies from one of our laboratories demonstrated that in mice, global genetic deletion of the inhibitory G protein Gα_i2_ (which mediates muscarinic influences on the heart) is associated with a reduced vERP, a prolonged corrected QT interval (QTc), and a predisposition to VT.^11^ There is also evidence that the protective vagal influence on cardiac electrical stability might be mediated by nitric oxide (NO), produced by neuronal NO synthase (nNOS).^12^, ^13^

Despite this evidence, there has been no attempt to study the central nervous mechanisms underlying parasympathetic antiarrhythmic influences. In this study, we aimed to identify a population of vagal preganglionic neurons that provide functional parasympathetic innervation of the ventricles and control ventricular excitability. Vagal preganglionic neurons projecting to cardiac ganglia are located in the brainstem. Neuronal tracing experiments in different species^14^, ^15^ have identified the dorsal vagal motor nucleus (DVMN) as one such population of neurons with long-latency C-fiber axons converging on cardiac plexuses.^14^ Because the activities of DVMN neurons appear to protect ventricular cardiomyocytes against acute ischemia/reperfusion injury,^16^ we hypothesized that functional ventricular innervation is provided by DVMN neuronal projections.

Autonomic dysfunction has emerging clinical importance contributing to the pathogenesis of Parkinson’s disease.^17^, ^18^ Parkinson’s disease is the second most common neurodegenerative disorder^19^ characterized by loss of dopaminergic neurons, with consequent motor impairment as well as DVMN dysfunction, which results in a host of autonomic abnormalities.^18^, ^20^ Aggregation of α-synuclein is a major molecular event in the development of the disease because of the toxicity of certain intermediate products of this process. Age-dependent decline of substantia nigra pars compacta synaptic function has been reported in α-synuclein–deficient mice.^21^ These changes appeared to be even more pronounced in triple-synuclein-null (αβγ^−/−^) mice that have been generated to limit functional compensation by other members of the synuclein family.^22^ In this study, we used αβγ^−/−^ mice to determine whether synuclein deficiency leads to a reduction in the activity of DVMN neurons and has an impact on the electrophysiological properties of the ventricle.

## Methods

All experiments were performed in accordance with European Commission Directive 2010/63/EU (European Convention for the Protection of Vertebrate Animals Used for Experimental and Other Scientific Purposes) and the UK Home Office (Scientific Procedures) Act (1986) with project approval from the respective institutional animal care and use committee.

### Cardiac electrophysiology

Cardiac pacing with extrastimulation was performed with a Grass S88 stimulator (Grass Instruments/Natus Neurology, Warwick, RI) as described in detail previously.^11^ Adult male Sprague-Dawley rats (weight 380–450 g), wild-type (WT) mice, and αβγ^−/−^ mice were anesthetized with urethane (1.3 g/kg IP), and an octapolar electrophysiology catheter (1.6F for rats and 1.1F for mice) was positioned in the right atrium and the right ventricle via the jugular vein or in the left ventricle via the right common carotid artery. For assessment of vERP, 10 paced beats (10 × S1) were applied with a cycle length of 85 ms in mice and 100 ms in rats, followed by a gradually shortened extra single paced beat (S2) until failure of ventricular capture. The maximum S1-S2 coupling interval was measured as the vERP. VT threshold (in rats only) was evaluated by application of 15 paced beats (15 × S1), with the cycle length shortened from 100 ms to 20 ms in 4ms increments (10–60 Hz burst pacing). One episode of VT was defined as at least 10 consecutive broad complex systoles (all of which were noted to undergo self-cardioversion). The definition of VT was similar to that in recent publications.^11^

For assessment of the atrioventricular node effective refractory period (AVNERP) in rats, 10 paced beats (10 × S1) with a cycle length of 141 ms were applied, followed by a gradually shortened extra single paced beat (S2) until failure of ventricular capture. The Wenckebach point was determined by shortening the cycle length (from 150 ms) until 2:1 block was observed. Sinus node recovery time (SNRT) was measured as the period between the last paced beat and the onset of the first P wave after 30 seconds of atrial pacing (cycle length 141 ms).

### Pharmacological study

To determine the presence of a tonic parasympathetic influence on cardiac electrical stability in the main experimental model used in the present study (rat anesthetized with urethane), systemic beta-adrenoceptor blockade was applied (atenolol 2 mg · kg^−1^ · h^−1^ IV), and the effects of sequential systemic muscarinic (atropine methyl nitrate 2 mg^−1^ · kg^−1^ · h^−1^ IV) and nNOS (7-nitroindazole [7-NI]; 20 mg/kg IP) blockade on AVNERP, SNRP, Wenckebach point, left and right vERP, and left and right VT were determined.

### Targeting vagal preganglionic neurons in the DVMN

Cholinergic vagal preganglionic neurons of the DVMN characteristically express the transcriptional factor Phox2 and can be targeted to express the gene of interest using viral vectors with Phox2 activated promoter (PRSx8). DVMN neurons were transduced with lentiviral vectors (LVVs) to express either the G_i_-protein–coupled *Drosophila* allatostatin receptor (AlstR) or enhanced green fluorescent protein (eGFP; control). Application of a natural ligand of AlstR, the insect peptide allatostatin, results in a highly selective, rapid, and reversible silencing of the DVMN neurons expressing AlstR.^16^

Male Sprague-Dawley rats (weight 50 g) were anesthetized with ketamine (60 mg/kg IM) and medetomidine (250 μg/kg IM). Adequate depth of surgical anesthesia was confirmed by the absence of a withdrawal response to a paw pinch. With the head fixed prone in the stereotaxic frame, the dorsal surface of the brainstem was exposed. DVMN neurons were targeted with 2 microinjections per side (0.25 μL; 0.05 μL/min) of a solution containing viral particles of PRSx8-AlstR-eGFP-LVV or PRSx8-eGFP-LVV. The injections were made at 0.5 mm rostral, 0.6 mm lateral, and 0.8 mm ventral and at 1.0 mm rostral, 0.8 mm lateral, and 0.6 mm ventral from the calamus scriptorius. Anesthesia was reversed with atipamezole (1 mg/kg IM). For postoperative analgesia, rats were administered buprenorphine (0.05 mg · kg^−1^ · d^−1^ SC) for 3 days and carprofen (4 mg · kg^−1^ · d^−1^ IP) for 5 days. One rat injected with PRSx8-AlstR-eGFP-LVV developed middle ear infection and was excluded from the study. Rats weighed 300 to 400 g at the time of the experiments.

Vagal preganglionic neurons transduced to express AlstR along the left and right extent of the DVMN were silenced by allatostatin infusion into the cisterna magna (as described previously^16^), and the effects of this treatment on vERP and VT threshold were determined in conditions of intact sympathetic tone.

In a separate experiment, conducted in conditions of systemic beta-adrenoceptor (atenolol) and muscarinic (atropine) blockade, atrial and ventricular pacing protocols were applied before and after acute bilateral silencing of the DVMN neurons by targeted microinjections of a potent GABA_A_ receptor agonist muscimol. Rats (n = 8) were anesthetized and instrumented for cardiac electrophysiology as described above, and the heads were fixed in the stereotaxic frame dorsal side up. An occipital craniotomy was performed to expose dorsal brainstem. A multibarreled glass micropipette (tip size 20–25 μm) was used for microinjections of a vehicle (saline) or muscimol (0.1 M; 40 nL; pH 7.4) into the left and right DVMN (coordinates: 0.3 mm rostral, 0.7 mm lateral, and 0.8 mm ventral from the calamus scriptorius). The injections were made using pressure over a period of 5 to 10 seconds and were monitored by use of a dissecting microscope with a calibrated micrometer disk.

### Triple-synuclein–null (αβγ^−/−^) mice

Young (6 months old) and aging (12–18 months old) αβγ^−/−^ and age-matched WT mice were used to determine whether synuclein deficiency is associated with a decline in DVMN activity and consequential changes in ventricular excitability. αβγ^−/−^ and WT mice were produced within the same breeding program (on the C57Bl/6J genetic background) as described previously.^22^ All 3 lines of single synuclein knockout mice were backcrossed with C57Bl/6J (Charles River, Wilmington, MA) for more than 8 generations before intercrossing to obtain double-knockout and finally αβγ^−/−^ mice. WT littermates obtained from intercrosses were used as controls.

### Derivations of QT interval

Needle electrodes were used to acquire a lead II electrocardiogram (ECG). The ECG was acquired for 15 minutes before programmed electrical stimulation and 15 minutes after allatostatin applications in rats expressing AlstR or eGFP in the DVMN, 15 minutes after saline or muscimol microinjections into the DVMN, and in αβγ^−/−^ and WT mice at baseline. In the pharmacological experiments, the ECG was acquired for 15 minutes before and after administration of each drug.

Three different approaches to deriving QTc were used because of the established controversies concerning its derivation in rodents.^23^, ^24^ QTc was derived by (1) Bazett’s formula: $QT_{\mathrm{BC}}=\mathrm{QT}÷\surd \left(\mathrm{RR}÷F\right)$ (where RR is in milliseconds, and F = 100 ms for mice and 150 ms for rats)^11^, ^25^; (2) Fridericia cubic root formula: $QT_{\mathrm{FC}}=\mathrm{QT}\times \sqrt[{3}]{\left(\mathrm{RR}÷F\right)}$ (where F = 100 ms for mice and 150 ms for rats); and (3) nomogram correction: $QT_{\mathrm{NC}}=\mathrm{QT}+0.384\times \left(150-\mathrm{RR}\right)$ for rats and QT + 0.156 × (100 − RR) for mice.^23^, ^24^ QTc was analyzed with the Tukey-Kramer multiple comparison test after 3-way analysis of variance to compare the effect of constructs (rats) or genotype (mice), allatostatin/vehicle (rats), age (mice), and QTc derivation formula. As a surrogate measure of ventricular repolarization that avoided rate dependency, raw QT measurements from vERP measurements were also analyzed.

### Humane endpoints

At the end of the experiments, the animals were euthanized with an overdose of pentobarbital sodium (200 mg/kg IP).

### Recording the activity of the DVMN neurons

Coronal brainstem slices (200 μm) were obtained from 12- to 18-month-old WT (n = 5) and αβγ^−/−^ (n = 4) mice terminally anesthetized with halothane. Brains were dissected in ice-cold low [Na^+^] solution containing 200 mM sucrose, 2.5 mM KCl, 28 mM NaHCO_3_, 1.25 mM NaH_2_PO_4_, 3 mM pyruvate, 7 mM MgCl_2_, 0.5 mM CaCl_2_, and 7 mM glucose (pH 7.4). After recovery at 34°C for 30 minutes in a solution containing 118 mM NaCl, 3 mM KCl, 25 mM NaHCO_3_, 1.2 mM NaH_2_PO_4_, 7 mM MgCl_2_, 0.5 mM CaCl_2_, and 2.5 mM glucose (pH 7.4), slices were kept at 34°C in artificial cerebrospinal fluid containing 118 mM NaCl, 3 mM KCl, 25 mM NaHCO_3_, 1 mM MgCl_2_, 2 mM CaCl_2_, and 10 mM glucose saturated with 95% O_2_ and 5% CO_2_ (pH 7.4).

Recording pipettes (3–6 MΩ) were pulled from thin-walled borosilicate capillaries, and recordings were performed in cell-attached mode with an EPC-9 amplifier and Pulse/Pulsefit software (Heka Elektronik, Lambrecht, Germany). Electrodes were filled with 120 mM K-gluconate, 5 mM HEPES, 5 mM BAPTA, 1 mM NaCl, 1 mM MgCl_2_, 1 mM CaCl_2_, and 2 mM K_2_ATP (pH 7.2). Recordings were performed in artificial cerebrospinal fluid saturated with 95% O_2_ and 5% CO_2_ at 28°C to 32°C. Currents were filtered at 1 kHz and digitized at 3 kHz. Mean action potential frequency was determined by taking the average frequency over a period of 3 minutes. Recordings were analyzed with Strathclyde Electrophysiology Software (WinEDR/WinWCP; University of Strathclyde, Glasgow, United Kingdom).

### Data analysis

Data are expressed as mean ± standard error. Interactions between groups or treatments were analyzed with analysis of variance with a Tukey-Kramer multiple comparison test, nonparametric Mann-Whitney *U* test, or paired Student *t* test as appropriate. DVMN neuronal recordings from each animal were averaged and analyzed with a Wilcoxon rank sum test for difference in medians.

## Results

### Parasympathetic influences on cardiac electrophysiology mediated by acetylcholine and nitric oxide are preserved under urethane anesthesia

**Figure 1:**
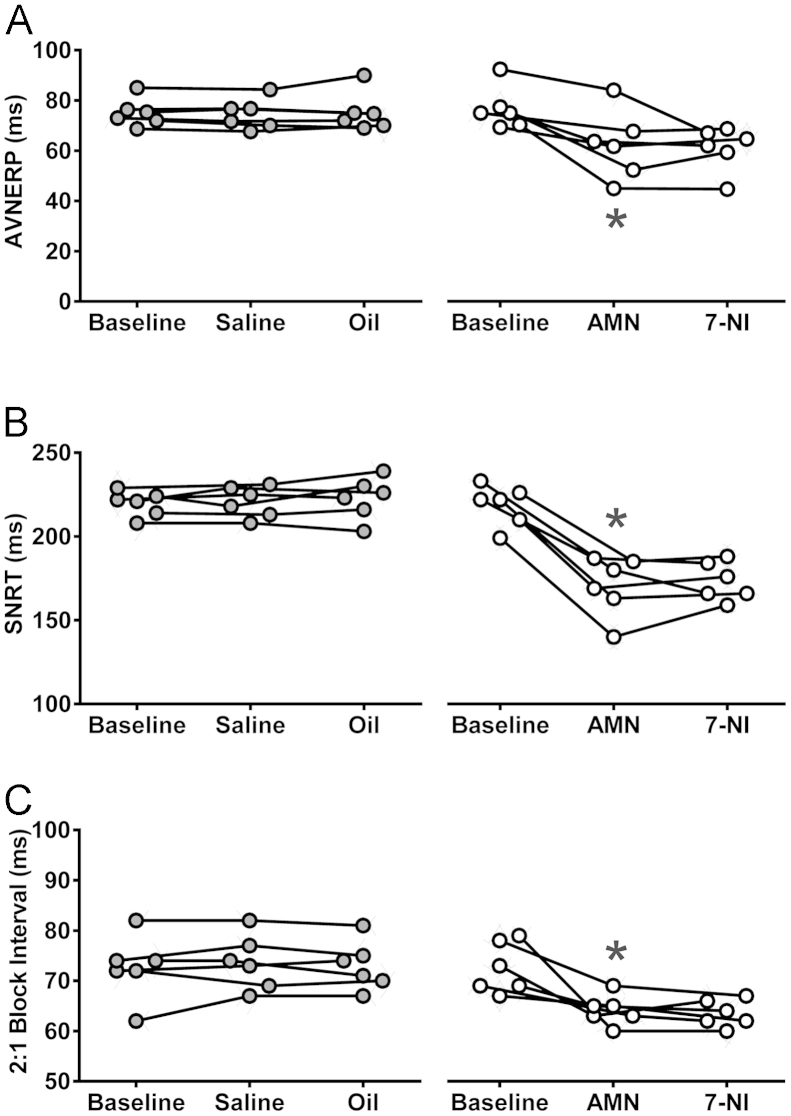
Tonic muscarinic influence on the electrical properties of nodal tissue. Summary data obtained in rats anesthetized with urethane in conditions of systemic beta-adrenoceptor blockade illustrating changes in atrioventricular node effective refractory period (AVNERP; **A**), sinus node recovery time (SNRT; **B**), and coupling interval required to achieve 2:1 heart block (**C**) after sequential systemic administration of atropine methyl nitrate (AMN) and neuronal nitric oxide synthase inhibitor 7-nitroindazole (7-NI) or respective control vehicles (saline for AMN and peanut oil for 7-NI). *Significant difference compared with baseline values (*P* < .05).

In conditions of beta-adrenoceptor blockade (atenolol), atropine administration reduced AVNERP (62 ± 6 vs 77 ± 3 ms at baseline; *P* = .003, Tukey-Kramer) (Figure 1A) and SNRT (171 ± 7 vs 219 ± 5 ms at baseline; *P* = .002, Tukey-Kramer) (Figure 1B) and lowered the S1 coupling interval required to achieve 2:1 heart block (64 ± 1 vs 73 ± 2 ms at baseline; *P* = .0008, Tukey-Kramer) (Figure 1C). No significant changes were observed after subsequent systemic nNOS blockade with 7-NI or during the course of the experiment in the group of time/vehicle control animals (Figure 1).

**Figure 2:**
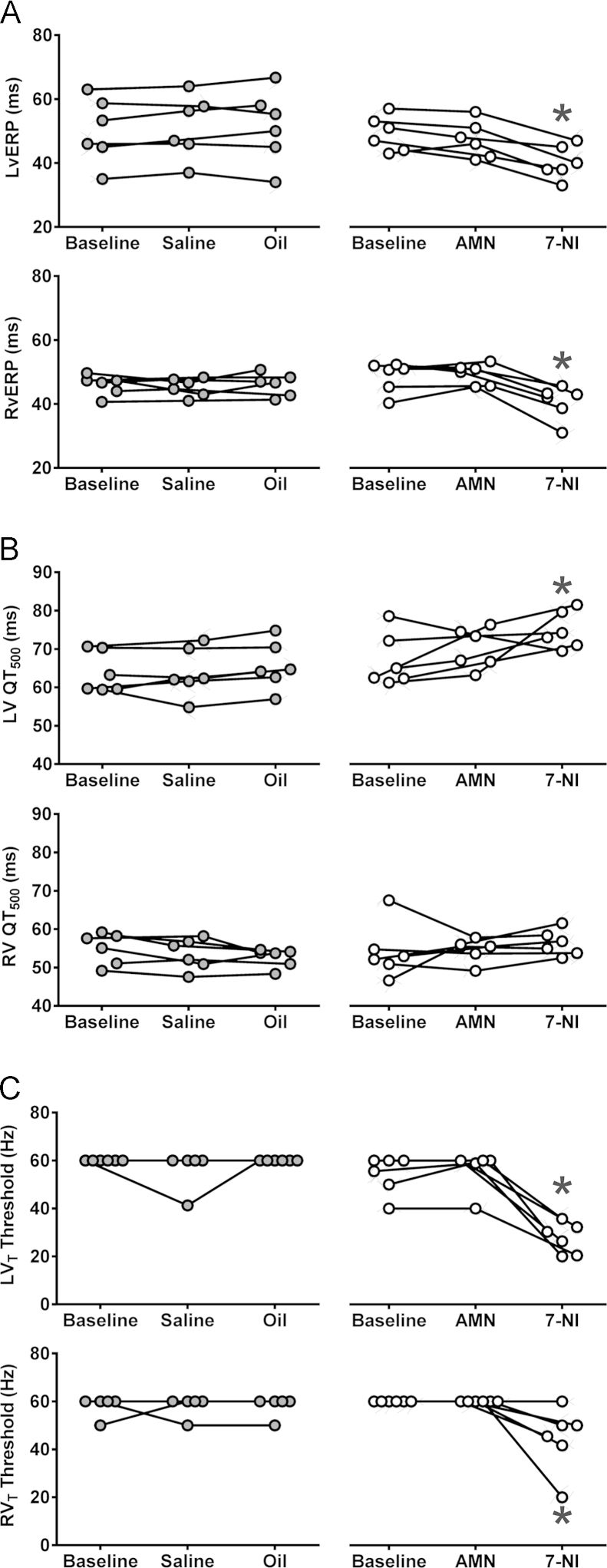
Tonic nitric oxide–mediated influence on the electrical properties of the ventricles. Summary data obtained in rats anesthetized with urethane in conditions of systemic beta-adrenoceptor blockade illustrating changes in left and right ventricular effective refractory period (LvERP and RvERP; **A**), left ventricle (LV) and right ventricle (RV) raw QT (QT_500_, measurements taken during vERP assessment protocol; **B**), and left and right ventricular tachycardia (LV_T_ and RV_T_) thresholds after sequential systemic administration of atropine methyl nitrate (AMN) and 7-nitroindazole (7-NI) or respective control vehicles (saline for AMN and peanut oil for 7-NI; **C**). *Significant difference compared with baseline values (*P* < .05).

In the ventricular pacing paradigm (in conditions of systemic beta-adrenoceptor blockade), atropine had no effect on left or right vERP or VT (Figure 2). Addition of systemic nNOS blockade was associated with a reduction of both left (40 ± 2 vs 49 ± 2 ms at baseline; *P* = .001, Tukey-Kramer) (Figure 2A) and right (41 ± 2 vs 49 ± 2 ms at baseline; *P* = .03, Tukey-Kramer) (Figure 2A) vERP. 7-NI administration produced a modest QTc prolongation measured during vERP assessment protocols in the left ventricle only (74 ± 2 vs 67 ± 3 ms at baseline; *P* = .02, Tukey-Kramer) (Figure 2B). This was concordant with the surface ECG data pooled from all atrial and ventricular pacing experiments, which revealed a modest prolongation in 2 derivations of QTc (Online Supplement Table 1). nNOS blockade also resulted in lowering of the left (28 ± 3 vs 54 ± 3 ms at baseline; *P* = .0002, Tukey-Kramer) (Figure 2C) and right (45 ± 6 vs 60 ± 0 ms at baseline; *P* = .001, Tukey-Kramer) (Figure 2C) VT thresholds.

### Effect of DVMN silencing on cardiac electrophysiology

**Figure 3:**
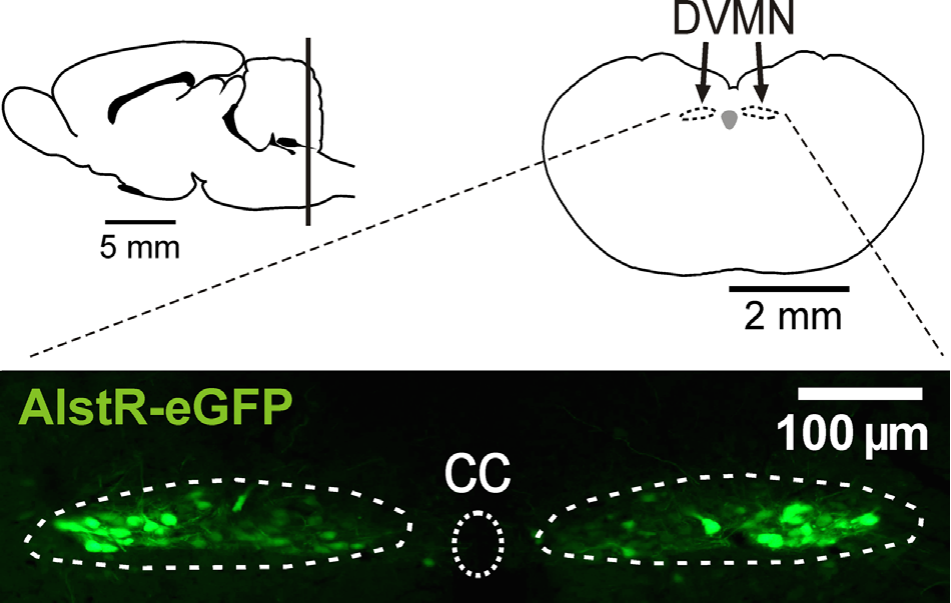
Schematic showing location of the dorsal motor nucleus of the vagus nerve (DVMN) in the rat brain. **Bottom:** Confocal image of coronal section of the rat brainstem, targeted to express allatostatin receptor (AlstR) in the DVMN. DVMN neurons are transduced to express AlstR-enhanced green fluorescent protein (eGFP). CC = central canal.

**Figure 4:**
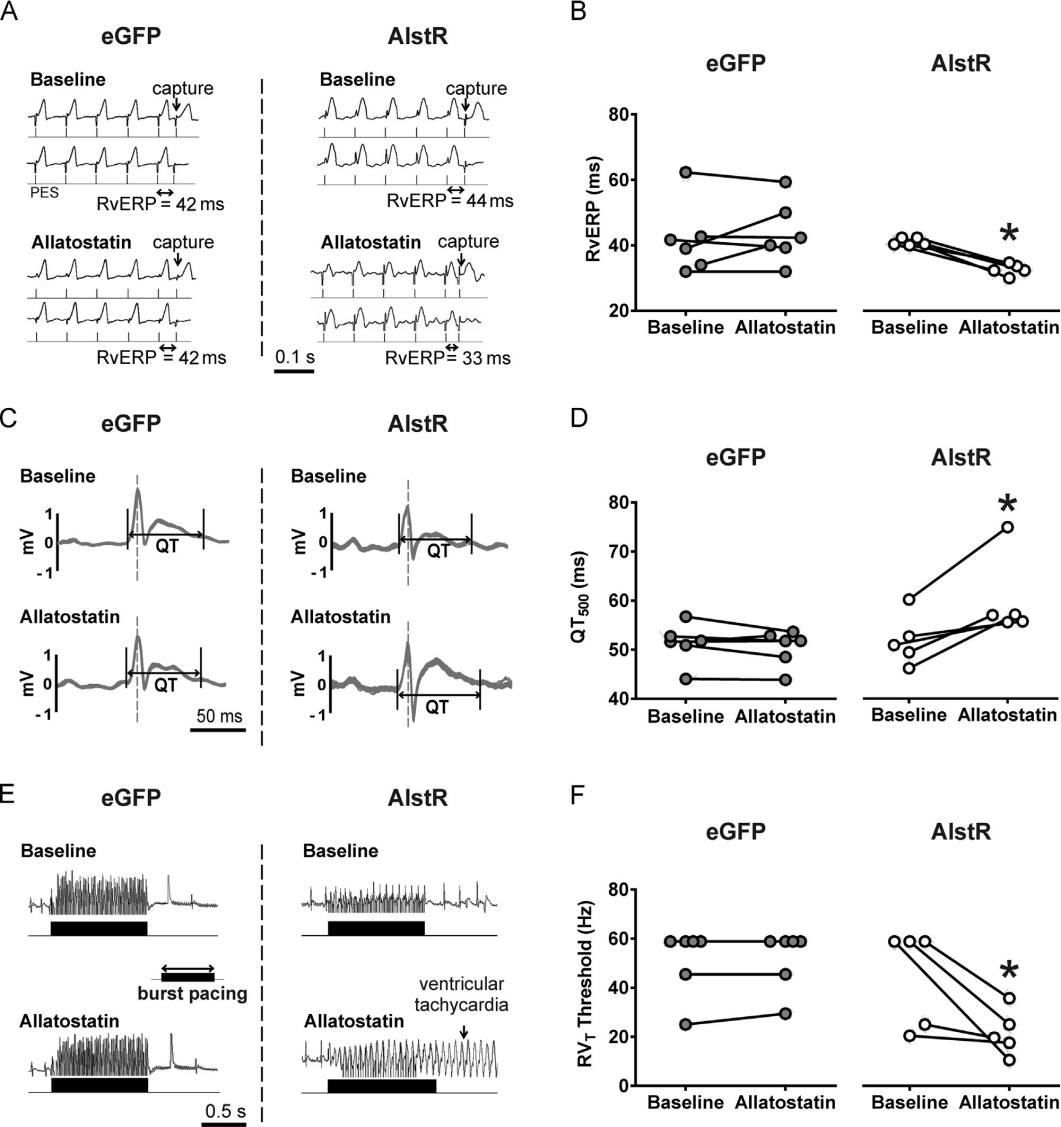
The effect of specific silencing of the dorsal vagal motor nucleus (DVMN) preganglionic neurons on ventricular excitability. **A:** Representative in vivo cardiac electrophysiology data produced by programmed electrical stimulation (PES) showing an example of right ventricular effective refractory period (RvERP) shortening in rats expressing the allatostatin receptor (AlstR) in DVMN after administration of allatostatin. **B:** Summary data illustrating RvERP shortening after inhibition of DVMN neurons. **C:** Representative signal-averaged electrocardiogram recordings illustrating QT prolongation in rats expressing AlstR in DVMN after administration of allatostatin. **D:** Summary data showing QT_500_ prolongation after inhibition of DVMN neurons. **E:** Representative in vivo cardiac electrophysiology data produced by burst pacing in increasing frequencies showing an example of a lower ventricular tachycardia threshold in rats expressing AlstR in DVMN after allatostatin application. **F:** Summary data illustrating decreased right ventricular tachycardia (RV_T_) threshold in rats after DVMN silencing. eGFP = rats expressing enhanced green fluorescent protein in DVMN. *Significant difference compared with baseline values (*P* < .05).

Application of allatostatin in rats expressing AlstR in the DVMN (Figure 3) resulted in vERP shortening (33 ± 1 vs 42 ± 1 ms at baseline; *P* = .002, Tukey-Kramer) Figure 4A and Figure 4B in the absence of heart rate changes (*P* = .8) (Online Supplemental Table 2). vERP reduction is known to be proarrhythmic in situations of both reentry and triggered activity. Analyses of averaged ECG waveforms revealed a prolongation of QTc according to all 3 correction formulas (*P* = .028, Tukey-Kramer) (Online Supplemental Table 2) (Figure 4C). Measurements obtained during the vERP assessment protocol also revealed QT prolongation in conditions of DVMN inhibition (60 ± 4 vs 52 ± 2 ms at baseline; *P* = .04, Tukey-Kramer) (Figure 4D). VT threshold (manifesting in this case as either monomorphic or polymorphic tachycardia) was lowered after DVMN inhibition (22 ± 4 vs 44 ± 9 Hz at baseline; *P* = .007, Tukey-Kramer) Figure 4E and Figure 4F. Application of allatostatin in rats expressing eGFP in the DVMN had no effect on vERP (42 ± 4 vs 44 ± 0.4 ms at baseline), VT threshold (51 ± 6 vs 52 ± 5 Hz at baseline), or QTc (Online Supplemental Table 2). Four rats expressing eGFP remained negative for VT (up to 60 Hz) throughout the course of the experiment. Three animals expressing AlstR in the DVMN that were negative for VT before allatostatin administration displayed reduced VT thresholds 30 minutes after application of the peptide.

**Figure 5:**
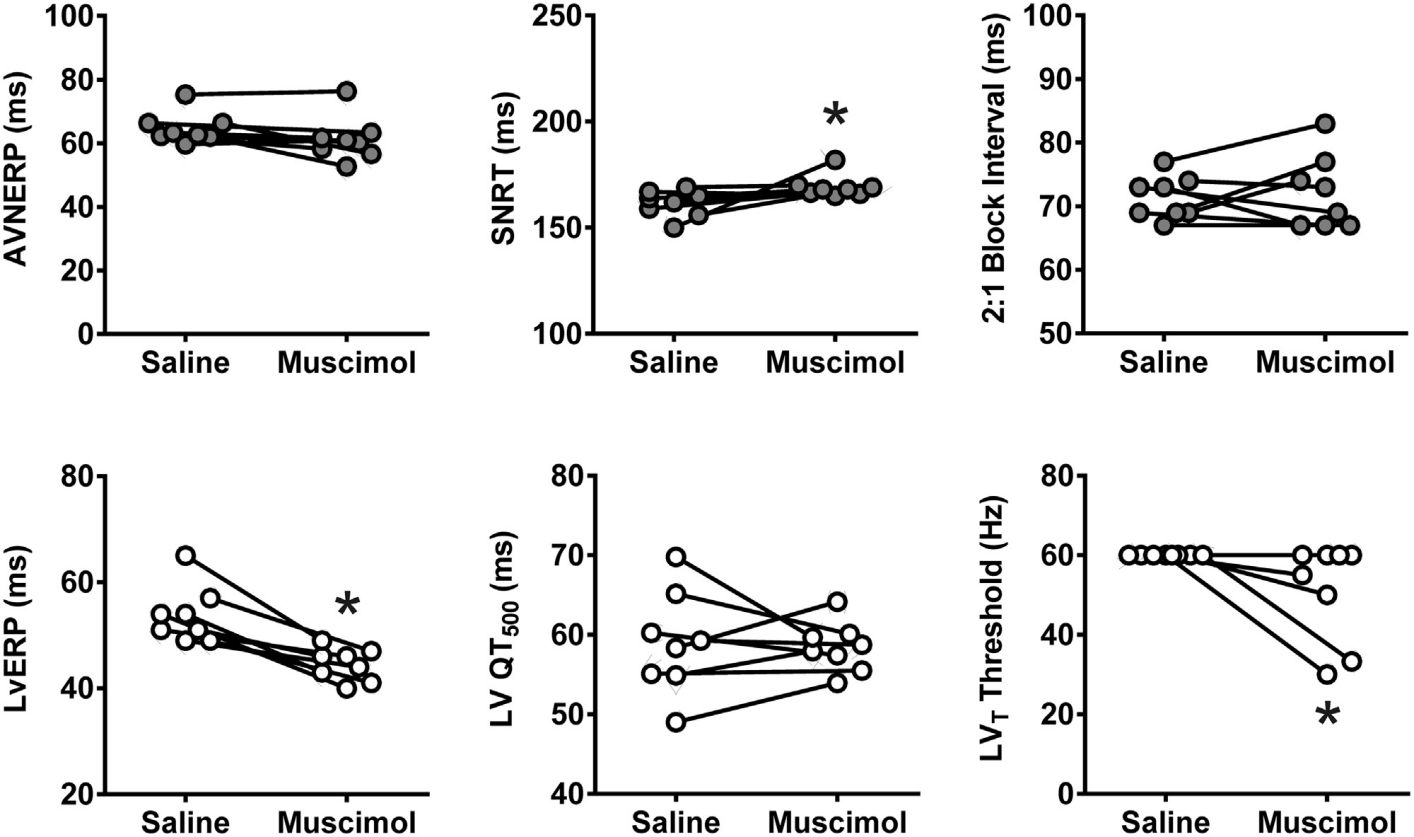
Effect of bilateral inhibition of dorsal vagal motor nucleus (DVMN) neurons by targeted microinjections of muscimol on the electrical properties of the heart in conditions of combined beta-adrenoceptor and muscarinic blockade. Summary data illustrating atrioventricular node effective refractory period (AVNERP), sinus node recovery time (SNRT), the coupling interval required to achieve 2:1 heart block, left ventricular effective refractory period (LvERP), left ventricular (LV) QT_500_, and LV tachycardia (LV_T_) threshold values after bilateral saline and muscimol microinjections into DVMN. *Significant difference compared with saline effect (*P* < .05).

In conditions of systemic beta-adrenoceptor and muscarinic blockade, inhibition of the DVMN neurons by bilateral microinjections of muscimol reduced left vERP (45 ± 22 vs 54 ± 2 ms at baseline; *P* = .02, Tukey-Kramer) and VT threshold (51 ± 4 vs 60 ± 0 Hz at baseline; *P* = .03, Tukey-Kramer) (Figure 5). In these conditions (beta-adrenoceptor and muscarinic blockade), DVMN inhibition resulted in a small increase of SNRT (169 ± 2 vs 162 ± 2 ms at baseline; *P* = .02, Tukey-Kramer) and had no effect on AVNERP, the coupling interval required to achieve 2:1 heart block, or QTc (Figure 5).

### Reduced activity of DVMN neurons in aging αβγ^−/−^ mice is associated with changes in ventricular excitability

**Figure 6:**
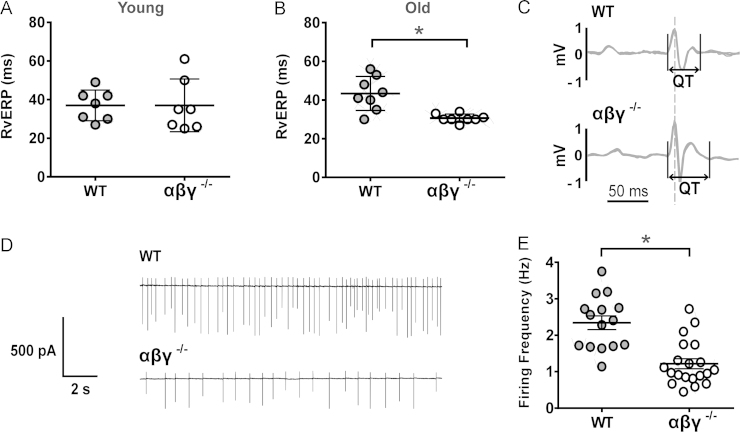
Reduced activity of dorsal vagal motor nucleus (DVMN) neurons in aging triple-synuclein–null (αβγ^−/−^) mice is associated with changes in ventricular excitability. **A:** At 6 months of age, there was no difference in right ventricular effective refractory period (RvERP) between wild-type (WT) and αβγ^−/−^ mice. **B:** At 12 to 18 months, αβγ^−/−^ mice had a significantly shorter RvERP than WT animals. **C:** Representative signal-averaged electrocardiography recordings illustrating QT prolongation in αβγ^−/−^ mice. **D:** Representative recordings of electrical activity of DVMN neurons in WT and αβγ^−/−^ mice. **E:** DVMN neurons of older αβγ^−/−^ mice have a significantly reduced firing frequency compared with age- and sex-matched WT counterparts. ^⁎^Significant difference between WT and αβγ^−/−^ (*P* < .05).

Six-month-old αβγ^−/−^ mice and their WT counterparts showed no significant differences in cardiac electrophysiology: both vERP (37 ± 5 vs 37 ± 3 ms) (Figure 6A) and ECG features, including QTc, were similar. In contrast, 12- to 18-month-old αβγ^−/−^ mice displayed a shorter vERP (31 ± 1 vs 43 ± 3 ms in WT mice; *P* = .002, Mann-Whitney *U* test) (Figure 6B) and a prolonged QTc (*P* < .05) (Online Supplemental Table 3) (Figure 6C). Electrophysiological recordings taken from DVMN neurons in acute brainstem slices showed a markedly reduced level of DVMN activity (~60%) in older αβγ^−/−^ mice (1.2 ± 0.1 vs 2.3 ± 0.2 Hz in WT mice; *P* = .04, Wilcoxon rank sum test) Figure 6D and Figure 6E.

## Discussion

The data obtained in the present study demonstrate that reduced activity of DVMN vagal preganglionic neurons has a significant impact on cardiac electrical properties, with changes consistent with the potential for ventricular arrhythmogenesis. Acute and highly selective silencing of DVMN neurons in a rat model was associated with vERP shortening and reduced VT threshold. The absence of changes in R-R and PR intervals, vERP shortening, and reduced VT threshold with no associated reduction in AVNERP or SNRT observed during DVMN silencing in conditions of beta-adrenoceptor and muscarinic blockade would suggest preferential innervation of the ventricles by DVMN neuronal projections.

Tonic parasympathetic influence on cardiac electrical stability is preserved in experimental animals kept under urethane anesthesia. In conditions of beta-adrenoceptor blockade, sequential systemic muscarinic and nNOS inhibition confirmed that vagal effects are mediated by the actions of acetylcholine and NO. Systemic atropine administration led to a reduction in AVNERP, SNRT, and coupling interval required to achieve 2:1 heart block and had no effect on vERP and VT. Systemic 7-NI treatment had no further effect on AVNERP, SNRT, and coupling interval but led to a significant reduction in both left and right vERP and VT. These data suggest that the vagal effects on cardiac electrophysiology are mediated by a muscarinic mechanism at the level of atria/nodal tissue and by NO at the level of the ventricles. The latter conclusion is concordant with the recent literature.^12^, ^13^

This NO-mediated parasympathetic influence on the heart appears to stem from the activity of the DVMN neurons. Indeed, when DVMN was silenced in conditions of combined muscarinic and beta-adrenoceptor blockade, only reductions in vERP and VT (and not in AVNERP or SNRT) were observed. We hypothesize that the cholinergic component originates from the population of vagal preganglionic neurons of the nucleus ambiguus, believed to provide the most important source of phasic parasympathetic control of the SA and AV nodes.^26^

Having previously demonstrated that synuclein deficiency results in age-dependent synaptic abnormalities,^22^ we used αβγ^−/−^ mice as a model of age-dependent autonomic dysfunction relevant to Parkinson’s disease. In aging αβγ^−/−^ mice, the reduced activity of DVMN neurons was found to be associated with changes in ventricular excitability similar to that observed in rats after acute DVMN inhibition.

In both models (rats and mice), we also measured and observed changes in the QTc interval. In general, we found a prolongation associated with vagal withdrawal; however, the magnitude of the recorded QTc changes varied depending on the experimental protocol. There are a number of considerations in interpreting these findings. The combination of shortened vERP and prolonged QTc is paradoxical; however, we have observed this previously in mice with global genetic deletion of Gαi_2_.^11^ We explained this by differences in the rate dependence of changes in the dynamics of repolarization. In addition, vERP and action potential duration are not always strictly correlated.^27^ Finally, there is some debate about the nature of the T wave in rodents and how it relates to ventricular repolarization.^28^

There are some limitations to our study. The rodents do not develop spontaneous arrhythmias, and we have not yet developed techniques for chronic DVMN inhibition in conscious rats that would allow longitudinal studies. Also, rodents are not an ideal model for studying disorders of cardiac repolarization, because the K^+^ currents affected are different in different species.^29^ Various murine models of cardiac arrhythmia have been developed, such as those with Na^+^ channel defects and autonomically driven arrhythmias associated with engineered defects in Ca^2+^ handling.^30^ However, the basic organizational, developmental, and signaling pathways between the brain and the heart are broadly conserved across small and large mammals, which gives the fundamental observations based on the reported data a clear translational relevance.^31^

In summary, in this study, we used a rat model to determine the effect of acute DVMN inhibition and a mouse model to study the effect of age-dependent decline in DVMN activity, relevant to the development of Parkinson’s disease. Both models revealed that reduced activity of dorsal vagal preganglionic neurons has a significant impact on cardiac electrical properties, with changes consistent with the development of a ventricular proarrhythmic phenotype. The vagal influence on ventricular excitability generated by the DVMN is likely to be mediated by NO. These findings are the first of their kind to provide a fundamental insight into the central nervous substrate that underlies functional parasympathetic innervation of the ventricles and highlight its vulnerability in neurodegenerative diseases.

**Clinical Perspectives**

Synuclein-deficient mice exhibit synaptic abnormalities with features similar to those observed in Parkinson’s disease and so appear to be an appropriate model of age-related loss of DVMN activity. Indeed, in patients with Parkinson’s disease and other neurodegenerative disorders, select neuronal populations such as the DVMN and substantia nigra pars compacta are affected most significantly. Loss of the DVMN activity would inevitably result in autonomic (parasympathetic) dysfunction and, as our data suggest, would be sufficient to have a significant impact on ventricular excitability. Neurons with long projecting axons with no myelination that require more energy for impulse propagation, like DVMN neurons, are the most susceptible,_ENREF_50 especially in the sporadic variants of Parkinson’s disease. Results obtained in the present study provide an insight into the central nervous substrate that underlies vagal control of cardiac ventricles and highlight its vulnerability in neurodegenerative diseases.

## Footnotes

Appendix Supplementary data associated with this article can be found in the online version at doi:10.1016/j.hrthm.2015.06.005.
