# Supplementary material for: Control of ventricular excitability by neurons of the dorsal motor nucleus of the vagus nerve
Source: Heart Rhythm. 2015 Nov;12(11):2285–93. doi: 10.1016/j.hrthm.2015.06.005 (PMC4631809; doi:10.1016/j.hrthm.2015.06.005)
Supplement: Supplementary file 1 — Supplementary Material [file mmc1.docx]

**SUPPLEMENTARY INFORMATION**

**Supplementary Table 1**

Parameters of ECG morphology recorded in rats anaesthetised with urethane in conditions of systemic beta-adrenoceptor blockade following sequential systemic administration of atropine methyl nitrate and neuronal nitric oxide synthase inhibitor 7-Nitroindazole or respective control vehicles (saline for atropine and peanut oil for 7-NI).

|  | **R-R (ms)** | **P-R (ms)** | **QT_BC_ (ms)** | **QT_FC_ (ms)** | **QT_NC_ (ms)** |
| --- | --- | --- | --- | --- | --- |
|  |  |  |  |  |  |
| **Time/Vehicle Controls**  (n=18) | |  |  |  |  |
| Baseline | 187 ± 3 | 45 ± 1 | 89 ± 1 | 106 ± 2 | 85 ± 2 |
| Saline | 186 ± 3 | 45 ± 1 | 90 ± 2 | 107 ± 2 | 86 ± 2 |
| Oil | 184 ± 3 | 44 ± 1 | 88 ± 1 | 105 ± 1 | 84 ± 2 |
|  |  |  |  |  |  |
| **Muscarinic/nNOS blockade**  (n=18) | | |  |  |  |
| Baseline | 184 ± 3 | 45 ± 1 | 89 ± 1 | 106 ± 1 | 85 ± 2 |
| AMN | 154 ± 3* | 48 ± 1 | 92 ± 1 | 94 ± 2 | 86 ± 2 |
| 7-NI | 170 ± 14 | 46 ± 2 | 96 ± 4* | 110 ± 14 | 110 ± 15* |
|  |  |  |  |  |  |

Values are presented as means ± SEM.

7-NI = 7-Nitroindazole; AMN = atropine methyl nitrate; QT_BC_ = QT correction using Bazett’s formula; QT_FC_ = QT correction using Fridericia’s formula; QT_NC_ = QT correction using the nomogram formula. *p<0.05 compared to baseline and control values.

**Supplementary Table 2**

Parameters of ECG morphology in rats expressing eGFP or AlstR in the neurones of the dorsal motor nucleus of the vagus nerve (DVMN) before and after administration of allatostatin

|  | **R-R (ms)** | **P-R (ms)** | **QT_BC_ (ms)** | **QT_FC_ (ms)** | **QT_NC_ (ms)** |
| --- | --- | --- | --- | --- | --- |
|  |  |  |  |  |  |
| **eGFP** (n=6) |  |  |  |  |  |
| Baseline | 149 ± 9 | 51 ± 3 | 67 ± 4 | 66 ± 4 | 70 ± 4 |
| Alst | 146 ± 8 | 49 ± 6 | 66 ± 4 | 65 ± 5 | 70 ± 4 |
|  |  |  |  |  |  |
| **AlstR** (n=5) |  |  |  |  |  |
| Baseline | 152 ± 6 | 50 ± 3 | 67 ± 4 | 67 ± 4 | 71 ± 4 |
| Alst | 145 ± 4 | 47 ± 2 | 83 ± 5* | 80 ± 6* | 85 ± 5* |
|  |  |  |  |  |  |

Values are presented as means ± SEM.

Alst = allatostatin (ligand for AlstR); AlstR = allatostatin receptor (animals transduced to express AlstR in the DVMN), eGFP = enhanced green fluorescent protein (animals transduced to express eGFP in the DVMN); QT_BC_ = QT correction using Bazett’s formula, QT_FC_ = QT correction using Fridericia’s formula; QT_NC_ = QT correction using the nomogram formula. *p<0.05 compared to baseline values considering gene-treatment-QT correction interactions.

**Supplementary Table** **3**

Parameters of ECG morphology in young and aging wild type and αβγ synuclein deficient mice

|  | **R-R (ms)** | **P-R (ms)** | **QT_BC_ (ms)** | **QT_FC_ (ms)** | **QT_NC_ (ms)** |
| --- | --- | --- | --- | --- | --- |
|  |  |  |  |  |  |
| **6 month old** | |  |  |  |  |
| WT (n=6) | 94 ± 1 | 32 ± 2 | 22 ± 2 | 20 ± 1 | 40 ± 18 |
| αβγ^-/-^ (n=6) | 108 ± 10 | 38 ± 3 | 21 ± 1 | 22 ± 1 | 21 ± 2 |
|  |  |  |  |  |  |
| **12-18 month old** | |  |  |  |  |
| WT (n=6) | 101 ± 4 | 33 ± 2 | 22 ± 1 | 22 ± 1 | 21 ± 1 |
| αβγ^-/-^ (n=6) | 92 ± 2 | 39 ± 3 | 26 ± 2* | 24 ± 2* | 26 ± 2* |
|  |  |  |  |  |  |

Values are presented as means ± SEM.

WT = wild type mice; αβγ^-/-^ = triple α,β,γ synuclein protein knockout mice; QT_BC_ = QT correction using Bazett’s formula; QT_FC_ = QT correction using Fridericia’s formula, QT_NC_ = QT correction using the nomogram formula. *****p<0.05 compared to WTs considering age-phenotype-QT correction interactions.
